# Effects of repeated hemolymph sampling from adductor muscles of relaxed Pacific oysters (*Magallana gigas*)

**DOI:** 10.1371/journal.pone.0333208

**Published:** 2025-09-22

**Authors:** Jingwei Song, Alexander A. Langley, Michael A. Banks, Bernarda Calla

**Affiliations:** 1 Department of Fisheries, Wildlife and Conservation Sciences, Coastal Oregon Experimental Station, Hatfield Marine Science Center, Oregon State University, Newport, Oregon, United States of America; 2 Pacific Shellfish Research Unit, United States Department of Agriculture, Agricultural Research Service, Newport, Oregon, United States of America; The University of Sydney School of Biological Sciences: The University of Sydney School of Life and Environmental Sciences, AUSTRALIA

## Abstract

Hemolymph is vital for bivalves, contributing to their innate immune system, nutrient transport, waste elimination, and hormone regulation. Yet, traditional sampling methods are often invasive or fatal to the organisms. In this study, we evaluated the effects of repeated, non-lethal hemolymph sampling on adult Pacific oysters (*Magallana gigas*). Five- year-old oysters were relaxed with magnesium sulphate, and hemolymph was extracted from half of the individuals, while the other half with relaxation served as controls. Sampling took place during gonad maturation in two groups: one laboratory conditioned (approximately three months in indoor tanks with controlled environment), and one naturally conditioned (approximately seven months at the bay in Yaquina Bay, Oregon, USA). Total mortalities ranged from 10% in the laboratory groups (after four samplings) and 22% (after seven samplings) in the natural groups; most of the mortalities took place after the last sampling. Sex ratio was similar between the sampled (66%) and control groups (63%) in the laboratory setting. In the natural setting, the final sex ratio was male biased in the group that was repeatedly sampled for hemolymph (58%) compared to the non-sampled controls (28%). Our findings highlight that repeated hemolymph sampling can be performed with minimal mortality, allowing non-lethal monitoring of hemolymph physiology over time.

## 1. Introduction

Hemolymph in bivalve mollusks is analogous to blood in vertebrates, consisting of immune cells (i.e., hemocytes) and plasma [1]. Hemolymph carries important proteins, minerals, nutrients, and hormones as well as hosting a diverse set of microorganisms including bacteria, protists, and viruses [2]. Studies of oyster hemolymph and its components have enhanced our understanding of bivalve innate immunity [3], disease dynamics such as that of Ostreid herpesvirus (OsHV-1) infection [4], effects of pollutants [5], importance of the microbiome on health [6], and sexual phenotypes [7].

The Pacific oyster, *Magallana gigas* (previously *Crassostrea gigas*), is the second most valuable aquaculture shellfish species by weight in the US, generating nearly 150 million dollars in sales annually [8]. The highly valued shellfish industry, however, faces challenges due to unpredictable mass mortalities usually associated with high seawater temperatures. Over the past two decades, mass mortalities in oyster farms have been reported in China [9], France [10], Australia [11], New Zealand [12], and in the United States [13,14]. Since hemolymph and its components are vital to the innate immune system in bivalves, there is continued interest in studying hemolymph in Pacific oysters.

Hemolymph sampling in oysters is often lethal or invasive [15–17]. Destructive sampling, such as shucking, prevents tracking changes in hemocyte composition, morphology, and function from individual animals over time. Invasive sampling, such as drilling through the shells or clipping the shell edges, are non-lethal but can result in high mortality. Few studies have attempted repeated sampling of hemolymph in *Magallana spp.* and *Crassostrea spp.*; all involving physical damage to the shell [3,6,18]. Mortality rates ranged from 15–20% [18] to as high as 41% [3]. Although oysters can repair their shells [19], repeated drilling or cutting of the shells makes these methods both labor-intensive and time-consuming.

A widely used non-lethal sampling method in bivalves involves the use of a muscle relaxant such as magnesium sulfate or magnesium chloride. Here, magnesium ions (Mg^2^+ ) can block the synaptic transmission of action potentials temporarily relaxing muscle tissue and causing the oysters to open [20]. This method was used for repeated sampling of mantle in *Crassostrea virginica* [21] and to sample gonads from *Magallana gigas* [22–24] with one study achieving nearly a 100% survival rate after three monthly sampling events [24].

The effect of combining magnesium-based relaxation with repeated sampling of hemolymph in Pacific oysters has not yet been tested. We conducted two separate experiments, one in a laboratory setting and one in a natural setting. The two experiments are fully independent of each other given the different experimental set up, duration, and number of samplings. While survival was the primary parameter assessed, we also quantified sex ratios as a proxy for sublethal physiological stress, since gonad maturation is an energy-intensive process and cohort sex ratios can reflect environmental and physiological conditions [25–27].

## 2. Materials and methods

### 2.1. Animal husbandry

Adult *Magallana gigas* (n = 200) were randomly selected from 5-year-old cohorts at the Oregon State University (OSU) Hatfield Marine Science Center, in Newport, Oregon [28]. The oysters were randomly assigned into Laboratory Control (LC), Laboratory Treatment (LT), Natural Control (NC), and Natural Treatment (NT) (n = 50 each). Oysters in both treatment groups (LT and NT) were individually labeled using Glue-On shellfish tags (Hinchinbrook, lnc.) and coral glue (Polyp Lab, lnc.). Each oyster was a biological replicate. All procedures were performed in compliance with OSU’s guidelines on animal research.

Oyster hatcheries use either naturally conditioned oysters or artificially conditioned oysters for spawning. The latter takes less time and can be performed year-round [29]. The “laboratory” groups (LC and LT) underwent a standard conditioning protocol used in oyster hatchery to induce gonad maturation [28]. Seawater was pumped from Yaquina Bay, Oregon and filtered down to 10 µm using a combination of sand filters and bag filters. The seawater was also UV sterilized before reaching the animal-holding tanks. Both laboratory groups were first kept at 6 °C in a chiller to induce oysters to resorb their gametes from the previous spawning season [29]. Starting on day 33, both groups were transferred to 130 L shallow flow through tanks (0.9 m x 0.96 m x 0.15 m). Here, the temperature was increased by 2 °C each day until it reached 20 °C, which was then maintained for 8 weeks. From the time the water temperature reached 20 °C, oysters were fed a mix of *Chaetoceros muelleri* (~60%) and *Tisochrysis lutea* (~40%) continually dosed into a common head tank and manifolds delivered algae-enriched seawater at a rate 2L/min and an inflow algal density of ~125,000 cells/mL. The National Center for Marine Algae (NCMA) provided the *C. muelleri* (CCMP 1316) and *T. lutea* (CCMP 463). Sterile cultures of both algae species were grown in F/2 medium (Pentair Aquatic Eco-Systems, USA) with a salinity of ~34 ppt and held at 21 °C in photobioreactors (Industrial Plankton Photobioreactors Inc, Victoria BC, Canada) with a light intensity of ~ 1,500 µmol m^-2^s^-2^ without a photoperiod. Algal concentrations were quantified using a hemocytometer with a Leica DM1000 LED microscope. The laboratory group experiment lasted 100 days.

The “natural” groups (NC and NT) were kept in cages (SEAPA, USA) hanging off a floating dock at the MBP repository in Yaquina Bay, Oregon (From November 2023 to May 2024, mean salinity = 25 ppt, mean water temperature = 8.7 °C) and brought onshore monthly for relaxation and hemolymph sampling from November 2023 to May 2024. This natural group experiment lasted 245 days.

### 2.2. Hemolymph extraction

Oysters were relaxed using magnesium sulfate USP (heptahydrate 100%) dissolved in a natural seawater/deionized water mix (1:1) at a final concentration of 8% (w/v) with constant aeration. Prior to relaxation, oysters were air exposed for 1 h at room temperature. This was demonstrated in our pilot trials to enhance the efficiency of shell opening. During relaxation, the laboratory group was maintained at the respective conditioning temperature: day 22: 6 °C; day 36: 18 °C; day 51: 20 °C; day 79: 20 °C, and the natural group was brought onshore and relaxed in the magnesium sulphate bath at room temperature (20°C).

**Fig 1:**
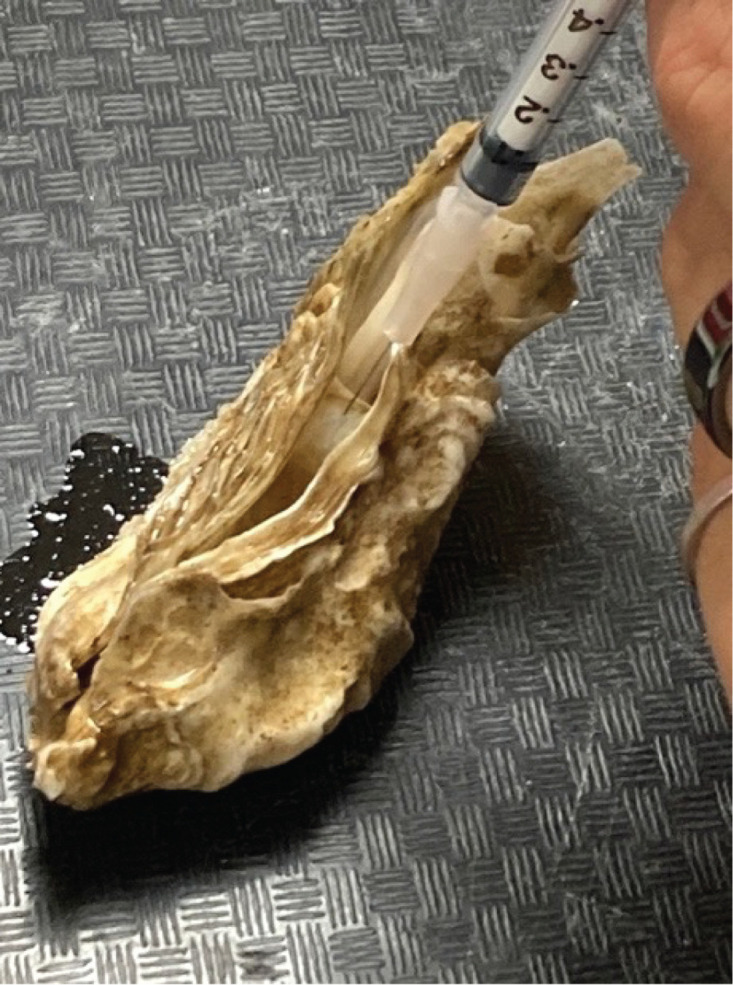
Hemolymph withdrawal from the adductor muscle of a relaxed Pacific oyster (*Magallana gigas*).

For hemolymph collection, oysters were individually removed from the magnesium bath after 18 h. Oysters were placed either horizontally with the cup side facing down or vertically in a dissecting tray (Fig 1), allowing hemolymph to concentrate on the distal side of the sampler due to gravity. A sterile 1 ml syringe and needle (30G, 25 mm, BD, USA) was used to collect the hemolymph, from each oyster. The needle was inserted into the adductor muscle perpendicular to the edge of the shell, and the plunger pulled gently to the 0.1 ml mark and left there for a few seconds until opaque hemolymph started flowing. After collection, each hemolymph sample was transferred to an individually pre-labeled 1.5 ml microcentrifuge tube. Efforts were made to obtain at least 100−200 µl of hemolymph from each oyster. To verify the presence of hemocytes, subsamples of the hemolymph (10 µl) were visualized under a light microscope on a hemocytometer. The extracted hemolymph was then immediately frozen in liquid nitrogen and transferred to a −80 °C freezer for long-term storage. Efforts were made to keep each oyster out of water for a maximum of 5 min, or after 5 needle insertions were made, whichever came first to minimize stress from prolonged air exposure while gaping.

Hemolymph from the LT oysters was collected on days: 22, 36, 51, and 79. Hemolymph from the NT oysters was collected every 30 days from November 2023 to May 2024 for a total of seven samplings. Oysters in LC and NC were relaxed following the same protocol as described above but no hemolymph was collected from them.

After sampling, all animals were first returned to a same flow-through recovery tank supplied with filtered seawater with conditions close to ambient in Yaquina Bay. Survival of oysters was checked after three hours by gently touching the upper shells to ensure shell closure before being returned to their respective holding tanks (laboratory group) or floating cages (natural group). Mortality was monitored daily for the laboratory group and monthly for the natural group.

### 2.3. Hemocyte density and oyster sexing

Oysters from the laboratory group were sexed 20 days after the last sampling (day 99 of the experiment). Due to logistical constraints, oysters from the natural group were sexed 2 months after the last sampling (on July 16 and 17, 2024). For sexing, animals were again relaxed, and micro-hematocyte capillary tubes (1.1 mm I.D., VWR) were used to take a sample of the gonad adjacent to the adductor muscle. The sample was placed on a glass slide with a drop of filtered seawater and viewed under a Leica DM1000 LED microscope at 100X-400X magnification. The sex of each oyster was recorded as either male, female, hermaphrodite, or N/A (if neither oocytes nor sperm were observed) [30]. To investigate whether sampling stress was reflected in the hemolymph, the hemocyte density in hemolymph samples taken from oysters that perished between the last sampling and sexing (N = 8, NT group) and from the ones that survived until sexing (N = 8, NT group) were compared. Samples from the final sampling date were retrieved from the −80 °C freezer, thawed on ice, and hemocyte counts were obtained using a hemocytometer with a Leica DM1000 LED microscope at 200X magnification equipped with a phase contrast objective. We did not measure hemocyte density in the LT group because of the low sample size of the perished oysters (N = 4) between the last sampling and sexing (Table 1).

**Table 1:** Sex of Pacific oysters in “laboratory” and “natural” groups. Control groups received relaxation only. Treatment groups received both relaxation and were sampled for hemolymph.

	Laboratory		Natural	
	Control	Treatment	Control	Treatment
Male	24	27	10	19
Female	14	14	26	14
Hermaphrodite	4	1	2	2
N/A	7	3	8	4
Total mortalities	1 (1)	5 (1)	4 (2)	11 (3)
Total	50	50	50	50
Percent males (%)	0.63	0.66	0.28	0.58

N/A = sex could not be determined. Number in parentheses show mortalities by the last hemolymph sampling event. Percent males (%) calculations excluded hermaphrodites, N/A, and mortalities.

### 2.4. Statistical analyses

The Kaplan-Meier estimates [31] were compared between the control and treatment groups using a log-rank test with the *survival* package v3.5-7 [32] in R [33]. The null hypothesis was that there was no difference in survival between the control and treatment groups (within laboratory or natural groups). Statistical significance was set at α = 0.05. A Chi-square test was used to evaluate the association between sex and treatment in the laboratory groups and natural groups (N/A and dead excluded, α = 0.05). Mann-Whitney U test [34] was used to compare hemocyte densities between the “dead” and “live” groups (α = 0.05).

## 3. Results

**Fig 2:**
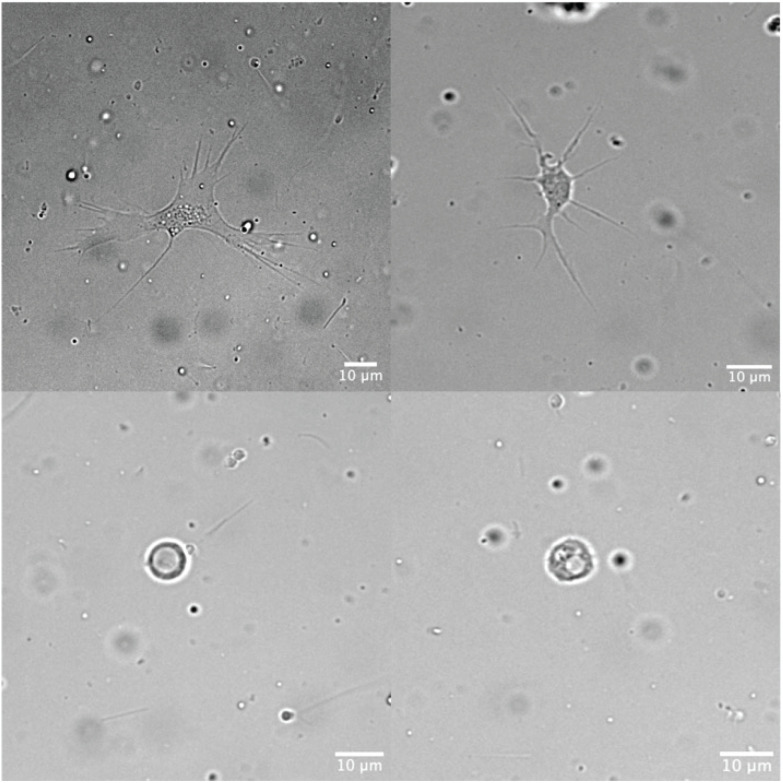
Live hemocytes of Pacific oyster (*Magallana gigas*). Top panels: granulocytes; bottom panels: hyalinocytes. Scale bars as shown.

The magnesium sulfate solution was effective in relaxing adult Pacific oysters across all groups. Most of the oysters remained open during the 5-minute sampling period, while a few slowly started to close during that time. Noticeably, one oyster in LT failed to open on two of the four sampling days. The amount of hemolymph collected ranged from 100–200 µl, but up to 2 ml of hemolymph could be obtained for some oysters. We also observed both granulocytes and hyalinocytes, in the extracted hemolymph samples (Fig 2). After being returned to their holding tanks, all oysters had closed their shells within three hours.

### 3.1. Laboratory groups

By the end of the fourth hemolymph sampling, only one mortality was observed in LC. In LT, one mortality was observed between the third and fourth sampling events, and four additional mortalities occurred during the period after the final hemolymph sampling but before sexing. The difference between survival probabilities was not statistically significant between LC and LT (**P* *= 0.1, χ^2^ = 2.7, df = 1). LC and LT had identical numbers of females (14 each), and a similar number of males (24 and 27, respectively) (Table 1). Sex was not associated with treatment (χ^2^ = 1.98, *P* = 0.37) in these laboratory groups.

### 3.2. Natural groups

**Fig 3:**
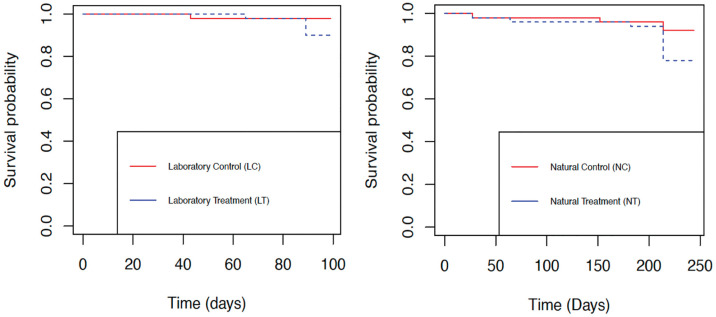
Cumulative survival plots of Pacific oysters (*Magallana gigas*) in a laboratory conditioned group (left) and a naturally conditioned group (right) subjected to repeated hemolymph withdrawal. Survival probability was not significantly different between LC and LT (*P *= 0.1, χ^2^ = 2.7, df = 1), nor between NC and NT (*P* = 0.06, χ^2^ = 3.6, df = 1).

Four mortalities were recorded in NC: two by the end of the seventh sampling event and two from the holding period before sexing. The highest total mortality rate was seen in NT (11 of 50, Table 1): Three mortalities were observed by the seventh hemolymph sampling event, and eight additional mortalities occurred during the holding period before sexing. The difference between survival probabilities was not statistically significant between NC and NT (*P* = 0.06, χ^2^ = 3.6, df = 1) (Fig 3). NC had a higher number of females than NT (26 and 14, respectively). Sex was associated with treatment (χ^2^ = 6.28, *P* = 0.04) in these natural groups. Hemocyte density ranged from 1.65 x 10^5^ to 1.59 x10^6^ per ml in hemolymph taken during the last sampling from oysters that died before the end of the experiment, and from 1.43 x10^5^ to 7.78 x10^5^ per ml in that from oysters that survived until the end of the experiment. Hemocyte densities were not significantly different between surviving and groups according to Mann-Whitney U test (W = 39.5, *P* = 0.4619).

## 4. Discussion

In this study, we demonstrated the effectiveness of a method for repeated hemolymph sampling in Pacific oysters (*Magallana gigas*). We evaluated the effects of magnesium-based relaxation for repeated hemolymph sampling on survival, sex ratios, and hemocyte density in an artificial, laboratory-controlled gonad conditioning setting, and in a natural setting where the animals experienced ambient fluctuations in water temperatures and food availability. Both laboratory and natural experimental contexts are presented here to offer insights that may be useful to researchers with varying access to animal husbandry resources, whether limited to laboratory or natural environments. The repeated relaxing and sampling resulted in minimal mortality in both settings, with extracted hemolymph volumes comparable to those from earlier invasive shell-damaging studies [6,18].

Mortality was low by the end of the last hemolymph sampling for all groups (2% — 6%) but increased to 10% and 22% by the final sexing. The low mortality result aligns with those of Suquet et al. [24], who observed only one mortality after three monthly samplings of gonad tissue in *M. gigas.* The total number of oysters sampled in that study was not reported, limiting our ability to make direct mortality rate comparisons.

We observed higher mortality after the final hemolymph sampling in both LT and NT compared to their respective controls. In general, sampling can cause physiological stress in animals [35], and hemolymph sampling has been shown to cause short-term stress in *M. gigas* within 24 h [36]. Hemocyte density has been correlated with physiological stress in bivalves [37]. The hemocyte density obtained here was lower than the one found in previous studies in bivalves [38,39], likely due to freezing and thawing without a cryoprotectant. We did not detect a significant difference in hemocyte density between the oysters that survived until sexing and those that perished between the last hemolymph sampling and sexing in the naturally conditioned group. Other hemolymph parameters, such as enzymatic and phagocytic activity, could potentially serve as more reliable indicators of sampling stress and subsequent survival. Previous studies [18,24] did not monitor mortality for an extended period post-experiment, which may have led to underreported mortality rates.

Environmental conditions and stress have been associated with sex outcomes in sequential hermaphroditic bivalves [29,40]. In *M. gigas*, nutrition, temperature, and photoperiod during gonad development could affect sex ratios [27,29,41,42]. We monitored sex ratios as a proxy for sublethal physiological stress of repeated sampling.

In the natural group, the energetic demands of repairing repeated tissue damage to the adductor muscles could have diverted resources from female gametogenesis—a process requiring more energy than male gametogenesis [25–27]—and might have contributed to the observed sex imbalance. While this result suggests that repeated hemolymph sampling in our natural treatment may have disrupted gametogenesis, particularly in females, we lack direct proof of a causal relationship. Manila clams (*Ruditapes philippinarum)* held in natural conditions showed no significant changes in hemocyte density or protein concentration between hemolymph samplings [39]. However, this does not eliminate the possibility of delayed effects, which remain unknown since the clams were not monitored beyond the experiment. Only the NC group had a female-biased sex ratio in line with expectations for a five-year-old cohort, suggesting that those oysters might have experienced the most favorable growing conditions without injuries from sampling.

In the laboratory groups, sex outcome was not associated with hemolymph sampling. A male-biased sex ratio was observed in these groups, not in line with the expectations for five-year-old cohort. Based on current knowledge, food availability [27], temperature [29,43], and age [43], could all affect sex ratios in Pacific oysters. The three-month conditioning period in the laboratory groups was shorter than the typical gametogenesis period for oysters in the Yaquina Bay, Oregon, which typically spans from March to September [44,45]. In addition, food availability and quality in the laboratory were likely inferior to what is normally available in the natural environment [39]. Thus, the shorter conditioning period, and food availability and quality likely negatively influenced the natural reproductive physiology of the oysters.

## 5. Conclusion

Hemolymph can be repeatedly sampled from the adductor muscles of relaxed adult Pacific oysters with minimal mortality, making this straightforward method potentially applicable to other bivalve mollusks. We demonstrate the method for both laboratory- and natural- gonad conditioning contexts, both of which are used in Pacific oyster hatcheries. Researchers planning to adapt this protocol to their study species should carefully evaluate experimental conditions including recovery setting, recovery duration, and sampling intervals among others.

## Supporting information

S1 File R scripts used in this study. (ZIP)
